# Obstetrics and Gynecology Emergency Department Activity during Lockdown in a Teaching Hospital, Hub Center, for COVID-19

**DOI:** 10.1155/2022/7557628

**Published:** 2022-09-05

**Authors:** R. Amadori, R. Buscemi, A. Desando, F. Grillo, V. Remorgida, D. Surico

**Affiliations:** Department of Obstetrics and Gynecology, University of Eastern Piedmont, Novara, Italy

## Abstract

**Background:**

The lockdown related to the SARS-CoV-2 pandemic has imposed profound changes in the interaction of the population with hospitals and emergency departments. The main aim of this research was to evaluate the impact of lockdown on the activity of obstetrics and gynecology emergency department (OGED) in a teaching hospital, hub center, for COVID-19.

**Methods:**

The study considers all visits to the OGED with their different triage color codes that represent the clinical severity of each case (from the most severe to the least one: red, yellow, green, white). Data were selected through the “PSNet” triage program and collected anonymously. We analyzed frequency distributions of the variables separately for each woman and calculated mean and standard deviations for continuous variables. We then analyzed the association between factors and outcomes for categorical variables (expressed as a number and percentage of the total) using the chi-square test (*χ*2). The level of significance was established with *p* < 0.05. Statistical analysis was performed using SPSS Statistics V20.0. Given the fact that the study has a retrospective observational nature and it is based on an anonymous routine database, approval by the Local Ethics Committee was not necessary.

**Results:**

The relative decrease of patients presenting to OGED in 2020 was −50.96%. The percentage of nonpregnant women was significantly lower in 2020 compared to 2019 (*p* ≤ 0.0001; Δ = −79.46%). Regarding the obstetric group, we saw an important decrease of visits in 2020 compared to 2019 (*p* < 0.0001; Δ = −40%). The prevalence of yellow codes was significantly higher in 2020 (Δ = +29.72%), while that of white (Δ = −61.58%) and green (Δ = −52.22%) codes was significantly lower (*p* ≤ 0.0001). Comparing the diagnoses at discharge, we could highlight significant reductions in 2020 for more than one diagnosis: bleeding (*p* ≤ 0.0001; Δ = −70.42%), pain (*p* ≤ 0.0001; Δ = −81.22%), urinary diseases (*p* = 0.004; Δ = −75.64%), and gastrointestinal diseases (*p* ≤ 0.0001; Δ = −87.50%).

**Conclusions:**

An evident change emerged in relation to the dynamics between the local obstetrical and gynecological population, and OGED resources. The COVID‐19 lockdown greatly reduced the rate of admission to OGED without time-related obstetric and gynecological complications. The reduction of admissions suggests a more appropriate use of the ED by patients that may inspire future policies for the implementation of emergency services.

## 1. Introduction

The main problem of an emergency department (ED) is overcrowding, increasing sanitary costs, decreasing patient satisfaction, decreasing morale of health workers, and even increasing morbidity and mortality according to the waiting time between the triage and the actual clinical examination [1–3]. Overcrowding and all the related problems are also open issues in the management of obstetrics and gynecology emergency department (OGED). Almost 1/3 of OGED visits are for nonurgent services [4,5].

The Italian government adopted several restrictive measures to implement social distancing and limit the spread of SARS-CoV-2 infection. On March 9 of 2020, a state of emergency was declared, commonly called “lockdown.” All nonessential activities such as office work, commercial activities, sports, and recreational activities were banned, including the free travel of people in and outside the whole country. Hospitals greatly modified their routine during and after lockdown by suspending all nonurgent outpatient consultations and planned surgery. All emergency and urgent consultations and procedures were guaranteed. Such strict measures were kept in place until May 4 of 2020.

The main objective of the study was to evaluate the impact of lockdown on OGED activity in a teaching hospital, hub center, for COVID-19. This hospital is placed in the second most afflicted Italian region in terms of the number of cases. It is less than 20 km away from Lombardy, the region with most cases in the country.

## 2. Materials and Methods

This is a retrospective monocentric study, conducted in the Department of Obstetrics and Gynecology at the “Maggiore della Carità” University Hospital, in Novara. The study compares all admissions to OGED during the lockdown period from March 9 to May 4 of 2020 with all the admissions of the same period in 2019. Our analysis starts from March 9 of 2020 because in that month Novara was one of the first Italian cities to be included in the red zone. The study considers all visits to OGED divided into triage color codes (red, yellow, green, and white). This hospital is a secondary care center, and it is active 24 hours a day; all patients with gynecological and obstetric symptoms refer directly to the OGED without going through the general ED. A multidisciplinary team works in OGED, including gynecologists, midwives, anesthetists, neonatologists, social workers, and nurses. Our OGED collaborates with the radiology and laboratory departments, and it is active 24 hours a day. The triage is performed by trained midwives. Then, according to the codes, the patients are evaluated by the physician on duty. In the hospital protocol, the management of triage codes is divided as follows: the red code means “emergency,” that is current impairment of a vital function of the woman or fetus at a gestational age ≥23 weeks; the yellow code means “urgency,” that is the threat of impairment of a vital function of the woman or fetus at a gestational age ≥23 weeks to be evaluated within 15 minutes; green code means “non-urgency,” that is service which can be postponed to be evaluated within 3 hours; white code means types of services similar to an outpatient service (Table1). Once the final diagnosis is made, the patient is dismissed from the OGED with a discharge color, often not the same color that was assigned at triage. In this work, we considered the discharge color code, not the admission one, because this is the one that is saved by the triage program. Data were extracted from “PSNet” (HiTech SPA, Software Engineering, Via di Campigliano 51, 50012 Florence, Italy) and collected anonymously. We calculated for each patient the minutes spent from admission to final decision (less than 30 minutes; from 30 to 60 minutes; from 60 to 90 minutes; from 90 to 120 minutes; more than 120 minutes). We analyzed all age groups (less than 35, from 35 to 50, from 50 to 65, and over 65) and all nationalities (Italian and others).

The study included all admissions, by considering all the pregnant women as obstetric patients (from 0 to 12 weeks, from 12 to 27 weeks, and from 27 weeks to term, first pregnancy or multiparous women) and also including puerperal patients in the gynecological patients. All patients who accessed OGED were included in the study results. There were no exclusions.

The symptoms reported by both the obstetrical and gynecological patients were as follows:

- Bleeding that includes spotting and abnormal uterine bleeding.
- Pain (abdominal, pelvic, and other generalized sites).
- Distal genital tract symptoms (vaginal or vulvar discomfort, including itching, redness, burning, and abscesses).
- Urinary symptoms (dysuria, strangury, oliguria, pollakiuria, and colic).
- Mammary symptoms (mainly redness, pain, or breast-feeding complications).
- Fever.
- Respiratory issues (cough and dyspnea).
- Gastrointestinal issues (diarrhea, vomit, and nausea).

Other symptoms complained of only by the gynecological group were as follows:

- Menstrual disorders.
- Pelvic organ prolapse.

The symptoms complained of only by pregnant patients were as follows:

- Uterine contractions (sporadic contractions and labor uterine contractions).
- PROM (suspected pre-labor rupture of membranes).
- Decreased fetal movements.
- Hypertension (hypertensive symptoms, also including headache, epigastric pain, visual disturbances, and tinnitus).
- Cardiac symptoms (mainly palpitations, tachycardia, lipothymia, syncope).

We then evaluated the patients for self-administered therapy before coming to triage.

We also collected the diagnosis at discharge, selected by the doctor at the end of the access. The discharge diagnosis for obstetrical and gynecological patients was as follows:

- Bleeding.
- Pain.
- Gastrointestinal diseases.
- Urinary diseases.
- Distal genital tract diseases.

Discharge diagnosis for gynecological patients was as follows:

- Postoperative complications.
- Menstrual disorders.
- Molar pregnancy and ectopic pregnancy.
- Mammary problems.
- Ovarian cysts.
- Sexual abuse.
- Benign neoplasm.
- Malignant neoplasm.

Diagnosis at discharge (obstetrical patients) was as follows:

- Miscarriage (inevitable abortion, incomplete abortion, septic abortion, complete abortion, and recurrent spontaneous abortion).
- Regular pregnancy (including uterine contractile activity without labor with home discharge).
- Labor/delivery.
- Premature rupture of membranes (PROM).
- Threatened abortion and threatened preterm labor.
- Hypertensive disorders.
- Cardiac problems.
- Decreased fetal movements.

Finally, patients were selected on the basis of the final decision:

- Discharging to home.
- Hospitalization.
- Leaving without being seen (LWBS) (after triage and before going to the examination room, or being examined but leaving before a final diagnosis was produced).

Considering the change in the number of patients accessing OGED during lockdown, our additional aim was to evaluate whether any time-related obstetric and gynecological complications occurred during that period. The complications could have developed before or during lockdown, and they could have become clinically evident after this period. For this reason, we decided to consider 60 days in addition to the ranges of time already taken into consideration (from March 9 to July 4) in both 2019 and 2020. The Italian National Health Service works on the principle that the local county will reimburse the hospital for the services provided. Therefore, each patient is dismissed with a coded diagnosis, coded diagnostic and therapeutic procedures. The codes are called International Classification of Disease-9th Revision (ICD-9). For each complication we looked up the corresponding ICD-9 code and we counted how many times this code was used during both analyzed periods. The data was given to us by the administrative office that stores the clinical notes and sends the ICD-9 codes to the region for the payment of the various services that the hospital provided. It should be considered that the ICD codes have not been recently reviewed; therefore, the distinction between moderate pre-eclampsia and severe HELLP disease still exists.

We considered the following maternal-fetal and gynecological complications:

- Small for gestational age (SGA) birth.
- Intrauterine growth restriction (IUGR).
- Stillbirth.
- Moderate preeclampsia.
- Severe preeclampsia/eclampsia/HELLP syndrome.
- Placental abruption.
- Preterm birth.
- Neonatal asphyxia.
- Miscarriage.
- Hemoperitoneum.

We analyzed frequency distributions of the variables separately for each woman and calculated mean and standard deviations for continuous variables. We then analyzed the association between factors and outcomes for categorical variables (expressed as a number and percentage of the total) using the chi-square test (*χ*2). The level of significance was established with *p* < 0.05. Statistical analysis was performed using SPSS Statistics V20.0 (IBM Corporation, Armonk, NY, USA). Given the retrospective observational nature of the study which is also based on an anonymous routine database, approval by the Local Ethics Committee was not necessary.

## 3. Results

The population involved in this study included a total of 1857 women admitted to the OGED in the same period of 2019 and 2020 (resp., 1246 and 611) (Table 2). The relative decrease of patients presenting to OGED was −50.96% (Table 2). The percentage of nonpregnant women was significantly lower in 2020 compared to 2019 (Table 2). We saw a significant decrease of the permanence times in OGED in 2020 mainly regarding stays that exceeded 120 minutes (Table 2). The prevalence of yellow codes was higher in 2020 (Table 2). Among the diagnoses of discharge, we could highlight reductions in bleeding, pain, urinary diseases, and gastrointestinal diseases (Table 3).

Among obstetrical patients, we found an important increase of the percentage of yellow codes and third-trimester pregnancy admission. The rate of hospitalization had a significant increase in percentage (Table 4).

About the amount of time in the OGED, it was clear that every code required less time to be managed, with a significant reduction of patients that waited more than 120 minutes. We found faster management within 30 minutes of all codes, in particular for the yellow one. We observed a reduction for all the admission symptoms and signs and for all the diagnoses at discharge. The decrease was more evident for bleeding, pain, and gastrointestinal and respiratory issues (Table 5).

In respect of the specific obstetrical symptoms, there was a percentage increase of patients that were admitted for uterine contractions and then hospitalized (Table 6).

We also noted an increased number of patients admitted for vaginal discharge, but a decreased number of patients with diagnosis of miscarriage, threatened abortion, or threatened preterm labor. We observed a reduction of gynecological patients discharged with diagnosis of pain and a rise in the percentage of distal genital tract issues (Table 7).

None of the time-related obstetric and gynecological complications showed significant changes despite the significant reduction in the number of visits during lockdown, even if the number of cases was too small and statistical significance was not demonstrated (Table 8)

## 4. Discussion

Unprecedented public health interventions were put into action to reduce the risk of SARS-CoV-2 transmission [6]. A sudden and sharp reduction in ED attendance began immediately after the spread of the first SARS-CoV-2 infection in Italy. Starting on February 21st, overall, ED attendance abruptly decreased by 41.8% in comparison to the previous year [7]. Elective surgery and outpatient visits were reduced; both medical and surgical wards were shifted to COVID-19 units; new supplementary sub-intensive and ICUs were created for COVID-19 patients [8]. The number of visits during lockdown decreased; in particular, for nonpregnant patients, the reduction was highly significant in comparison to previous data [9]; that reduction could be due to fear of contagion, transportation limitations, fear of getting infected, and increased use of telemedicine. Moreover, visits to private doctors' offices decreased [10,11].

During the last twenty years, OGEDs have been utilized for non-emergent medical care more and more, especially during pregnancy [4]. Emergency care settings in the US treat approximately 750000 patients annually for chief complaints related to gynecology and obstetrics. Interactions with the healthcare system during pregnancy and the postpartum period represent opportunities for health promotion [12], preventative health screenings [13], and engagement in long-term behavioral changes to reduce the future risk [14]. Some women have multiple barriers to outpatient follow-up, including lack of childcare, difficulties in accessing care, and lack of understanding about the long-term health risks associated with pregnancy complications [15]. These conditions may increase women's reliance on OGED settings [16]. This aspect could explain why in the lockdown period there was a reduction in the access of Italian patients, while this reduction was not equally present in patients with other origins, as also shown in another study [9].

The reduction of improper visits allowed the staff to attend to the real emergency codes, such as red and yellow, within the recommended time. Prompt ED service is expected when patients present to an emergency room with urgent health problems [17]. However, various factors can increase the waiting time for access to health services and also can prevent timely intervention for patients consulting emergency rooms [18]. As a result, the probability of serious complications, such as disability and death, has increased [19]. It is difficult to prevent emergency room congestion and overcrowding because patients, who do not require emergency care, also consult ED [20]. Extended ED waiting times result in a longer duration of pain experienced by patients, negative health effects, and reduced patient satisfaction [21]. Moreover, because of increased waiting times, growing numbers of patients leave the emergency room without receiving medical care [22]. As expected, the waiting time increases with increasing numbers of patients in the emergency room and reduces with decreasing numbers of patients consulting the emergency room [23]. Moreover, ED visits are often less efficient and less equipped for longitudinal care of chronic medical diagnoses [24]. In 2019, the percentage of LWBS was 8.2%; during 2020 lockdown period, it was 0.3%, with a significant decrease of −98%. LWBS is a major concern for physicians, healthcare providers, and hospitals [22]. The LWBS population, varying from less than <1% to more than 10% of all triaged patients (depending on the ED), has been considered in the literature as a shortfall in healthcare access, as these patients do not receive the care they originally sought [25]. Some studies suggest that LWBS rates may be an expression of the patient's level of safety as some patients who did not receive medical treatment had avoidable health outcomes. Conversely, it may also be hypothesized that the decision to LWBS reflects a lower-acuity complaint that has been resolved or will be resolved without medical intervention [26]. LWBS patients are high ED utilizers, accounting for nearly three times the number of visits in comparison to the general ED patients. They could represent a patients' population with persistent lack of access to reliable outpatient healthcare options or to adequate management of chronic pain and psychiatric conditions.

During our research, we saw an increase in the percentage of hospitalizations. This could be related to a reduction in less severe cases with a consequent proportional increase in situations of greater complexity. In support of this hypothesis, there was an increase in the percentage of yellow codes and a decrease in green and white codes. Moreover, a previous study on gynecological visits suggests that, during the COVID-19 lockdown, real emergencies have been screened from more deferrable cases. This led to a proportional increase of the number of hospitalized women, especially for emergent surgeries, and a decrease of the number of women discharged from the ED [27].

Pain seems to be the reason for which the most substantial reduction in OGED visits has occurred. This leads us to hypothesize that this symptom is considered by patients to be less alarming than bleeding, causing less discomfort, compared to vaginal infections.

The evidence of less visits to OGED for nonspecific symptoms suggests that the OGED was used more appropriately, due to the reduction of overcrowding, a decrease of inappropriate visits, and shorter waiting times.

For a more appropriate use of OGED, some changes could be useful: firstly, implementation of local health services to manage nonspecific symptoms in pregnancy; secondly, a more clear communication regarding how to manage pregnancy related symptoms during planned check-ups; thirdly, more expensive bills for accessing OGED for nonurgent reasons. This could allow less crowding and shorter waiting times, with staff being at ease during working hours.

One unexpected result, which amazed us, was the reduction in patients who tried to take medication, for example, a simple painkiller, before coming to the emergency room. In this regard, the hypotheses are the following: firstly, there was an increase in anxiety, so patients felt that it is safer to go to ED; secondly, it was more difficult to access a pharmacy and telephone advice of a doctor; thirdly, in the first months of the pandemic, fake news regarding the use of nonsteroidal anti-inflammatory drugs was published online [28].

As for second aim of our study, there were no differences between the time related to maternal-fetal or gynecological complications in the two periods under study, although there was a significant reduction in the number of visits for obstetric and gynecological reasons. In a previous published study, we demonstrated that there were no significant differences either for antepartum or intrapartum complications [29]. We found a high score for anxiety and depression, although it cannot be compared to the same score on the same population before the pandemic [30]. The high level of anxiety and depression we found is consistent with other studies [31,32]. The high prevalence of anxiety and depressive symptoms in pregnant women and new mothers should be a public health issue; in addition, screening for perinatal depression and anxiety should be considered during a pandemic. Data related to changes in obstetric or gynecological complications during lockdown are extremely conflicting [33].

An Italian study showed an increase of about three times in stillbirths, a decrease in the percentage of late preterm births, and an increase in full-term births. There was a nonsignificant increase in very preterm births and a nonsignificant reduction in caesarean sections [34].

Dell'Utri et al. showed a significant increase in the incidence of stillbirth, which did not occur in our setting. This could be explained as follows: either their catchment area is more extended than ours, or in Lombardy the percentage of sick professionals was higher than in Piedmont, and therefore our patients were able to contact their physicians for advice with less difficulty [9]. A Spanish study did not find any link between prematurity and lockdown, nor between stillbirths and lockdown [35]. A reduction in the rate of preterm birth was observed in France and Denmark [36]. Another study showed a decrease in iatrogenic preterm births during the initial COVID-19-related lockdown in the Netherlands in singletons [37]. An Iranian study showed, on the one hand, no significant differences in stillbirth rates or pregnancy complications (including preeclampsia, pregnancy-induced hypertension, and gestational diabetes) and, on the other hand, a decrease in preterm births and low birth weight in the pre- and intra-pandemic periods [38]. In Jordan, there were no significant differences in preterm birth and stillbirth rates, neonatal mortality, or perinatal mortality before and during the COVID-19 lockdown, but there was a significantly lower incidence of extreme low birth weight (ELBW) infants (<1 kg) during the COVID-19 lockdown period than that before the lockdown [39]. In India there was a reduction of 45.1% in institutional deliveries, a percentage point increase of 7.2 in high-risk pregnancy, and a 2.5-fold rise in admission to the intensive care unit of pregnant women during the pandemic. One-third of women had inadequate antenatal visits. The main reasons for delayed health-seeking were lockdown and fear of contracting infection, resulting in 44.7% of pregnancies with complications [40].

The major strength of our study is that we analyzed in detail and compared the symptoms for which patients, both obstetrical and gynecological, visited OGED by examining the variations of the assigned triage codes.

### 4.1. Study Limitations

The retrospective collection of patient data could have been biased by possible errors in the recording of data. The definition of the final diagnoses was highly heterogeneous in the electronic records. Although the definition of the principal complaint at triage was consistently quite homogeneous, it is also possible that the triage midwifes interpreted the patients' complaints in a different way. We chose not to analyze in this study the data relating to the activity of the delivery room, because the complexity of the variations requires a separate study, which is now under review. We describe what happened during the outbreak in Piedmont. It seems reasonable to speculate that this small-scale scenario could repeat itself in other, similar epidemiological contexts.

## 5. Conclusions

An evident change emerged in relation to the dynamics between the local obstetrical and gynecological population, and OGED resources. The COVID-19 lockdown greatly reduced the rate of admission to OGED, and this reduction suggests a more appropriate use of OGED that may inspire future policies for the implementation of emergency services. The reduction in the admission rate did not lead to an increase of time-related obstetric and gynecological complications, but further studies are needed to evaluate this.

## Figures and Tables

**Table 1:** Subdivision of gynecologic and obstetric complaints into triage color codes.

^Symptoms^	^Yellow^	^Green^	^White^	^Exceptions (associated symptoms)^	
^*Gynecologic complaints*^					
^Abdominal pain^	^Acute (unbearable pain that wakes the patient from sleep; the woman is completely focusing on it)^	^Moderate (difficult to bear, not allowing normal daily activities)^	^Mild (bearable, not interfering with normal daily activities)^	^If abdominal pain is associated with any of the following symptoms, yellow code is automatically assigned:^	
				^- Perspiration^	
				^- Nipple discharge^	
				^- Faintness^	
				^- Abdominal tenderness^	
				^- Vomit^	
				^- Fever^	
^Mammary issues^		^- Pain^	^Without symptoms or mild pain^	^If mammary issues are associated with any of the following symptoms, green code is automatically assigned:^	
		^- Flushing^		^- Fever^	
		^- Bleeding nipple^		^- Reported hematuria^	
		^- Nipple discharge^					
^Urinary symptoms/ distal genital tract pain^		^Severe (not allowing normal daily activities)^	^Moderate or mild (not interfering with normal daily activities)^		
^Post-op fever^	^Fever >= 38°^	^Fever < 38°^			
^Vaginal bleeding^	^Severe (more than one drenched pad per hour, clothes wet through)^		^Mild (full pad) or only spotting^	^If vaginal bleeding is associated with any of the following symptoms, green code is automatically assigned:^	
				^- Abdominal pain^	
				^- Fever^	
				^- Foul smell^	
				^- Faintness^	
				^- Pallor^	
^*Obstetric complaints*^					
^Symptoms^	^Red^	^Yellow^	^Green^	^White^	^Exceptions (symptoms associated)^
^Other complaints^		^Syncope (even past)^	^Diarrhea^	^- Earache^	^If other complaints are associated with any of the following symptoms, green code is automatically assigned:^
				^- Toothache^	^- Acute pain^
				^- Pharyngodynia^	^- Fever^
				^- Cough^	
				^- Constipation^	
				^- Nosebleed^	
				^- Hemorrhoids^	
				^- Weakness^	
				^- State of anxiety^	
^Headache^	^Acute (unbearable)^		^Moderate (difficult to bear, not allowing normal daily activities)^	^Mild (bearable, not interfering with normal daily activities)^	^If headache is associated with any of the following symptoms, yellow code is automatically assigned:^
					^- Pallor^
					^- Faintness^
					^- Visual disturbance^
					^- Vomit^
					^- Fever^
					^- BP >140/90 mmHg^
^Uterine contractions^	^Regular rate: 2–4 in 10 minutes with irrepressible need to push, stage 2 of labor^	^Regular rate: 2–4 in 10 minutes with no reported need to push, active phase of stage 1 of labor^	^Regular rate: <=1 in 10 minutes, prodromal stage^	^Irregular, sporadic, mild contractions (pre- labor)^	
^Convulsions^	^Convulsions (even past, recent, or brief)^				
^Mammary issues^			^- Pain^		
			^- Redness^		
			^- Bleeding^		
^Abdominal-pelvic pain^	^Acute (unbearable pain that wakes the patient from sleep; the woman is completely focusing on it)^	^Moderate (difficult to bear, not allowing normal daily activities)^	^Mild (bearable, not interfering with normal daily activities)^		^If abdominal-pelvic pain is associated with any of the following symptoms, yellow code is automatically assigned:^
					^- Perspiration^
					^- Pallor^
					^- Faintness^
					^- Abdominal Guarding^
					^- Vomit^
					^- Fever^
^Lumbosacral pain^	^Acute (unbearable pain that wakes^ ^the patient from sleep; the woman is completely focusing on it)^		^Moderate (difficult to bear, not allowing normal daily activities)^	^Mild (bearable, not interfering with normal daily activities)^	^If lumbosacral pain is associated with any of the following symptoms, yellow code is automatically assigned:^
					^- Walking or standing difficulty^
					^- Vomit^
					^- Fever^
					^- Perspiration^
					^- Pallor^
					^- Bleeding^
^Urinary/vaginal pain/burning^			^Severe (not allowing normal daily activities)^	^Moderate or mild (not interfering with normal daily activities)^	^If urinary/vaginal pain/burning is associated with any of the following symptoms, green code is automatically assigned:^
					^- Fever^
					^- Reported hematuria^
^Fever^		^Fever >= 38°^	^Fever < 38°^		
^Allergic phenomena^	^- Severe allergic phenomena^	^- Dyspnea^	^Urticaria-like^		
	^- Allergic shock^	^- Glottic edema^			
^Hypertension^	^Cyanosis^	^Severe hypertension (>=160/110)^	^Hypertension >=140/90–<160/110 with no associated symptoms^	^Hypertension <140/90 with no associated symptoms^	^If hypertension is associated with mild headache, green code is assigned^
					^If hypertension is associated with any of the following symptoms, yellow code is automatically assigned:^
					^- Persisting headache^
					^- Persistent epigastric pain^
					^- Visual disturbances^
					^- Dyspnea^
					^- Reduction of fetal movements above 23 weeks GA^
^No visualisation/perception of FHR <23^ ^weeks^	^Severe bleeding^	^- Pelvic pain/contractions^	^With no associated symptoms^		
		^- Moderate bleeding^			
^No visualisation/perception of FHR >= 23 weeks^	^- Bleeding^	^With no associated symptoms^			
	^- Contractions^				
	^- Acute abdominal/pelvic pain^				
	^- Uterine tetany^				
^No perception/reduction of fetal movements^		^>= 23 weeks of GA^		^< 23 weeks of GA^	
^Nausea and vomit^		^- Nausea and/or vomit >= 24 weeks GA^	^-Nausea and/or vomit with no associated symptoms^		
		^- Insulin-dependent diabetes^	^- Nausea and/or vomit < 24 weeks GA^		
		^- BP>140/90^			
		^- Headache^			
^Oligohydramnios^		^Oligohydramnios >=23 weeks^		^Oligohydramnios < 23 weeks^	
^Bleeding < 23 weeks GA^	^Severe (more than one drenched pad per hour, clothes wet through with associated symptoms)^		^- Moderate (period- like amount or with clots)^		F09F ^If bleeding < 23 weeks is associated with any of the following symptoms, yellow code is automatically assigned:^
			^- Mild, spotting with no associated symptoms^		^- Moderate pelvic pain^
			^- Recent bleeding^		^- Moderate lumbar pain^
					^- Dizziness^
					^- Nausea^
					^If it is associated with mild pelvic pain, green code is automatically assigned^
^Puerperal bleeding^		^Severe (more than one drenched pad per hour, clothes wet through)^		^Moderate (period-like amount or with clots)^	^If puerperal bleeding is associated with any of the following symptoms, green code is automatically assigned:^
					^- Abdominal/pelvic pain^
					^- Fever^
					^- Foul Smell^
					^- Dizziness^
					^- Pallor^
^Bleeding >= 23 weeks GA^	^- Severe (more than one drenched pad per hour, clothes wet through)^		^- Mild, spotting with no associated symptoms^		^(i) If bleeding >= 23 weeks GA is associated with any of the following symptoms, yellow code is automatically assigned:^
	^- Moderate (period-like amount or with clots)^		^- Past recent bleeding^		^- Pallor^
					^- Dizziness^
					^- Cold sweat^
					^- Abdominal/pelvic pain^
					^- Lumbosacral pain^
					^- Uterine tetany^
					^- Mild, long-lasting contractions^
^Vaginal discharge (not bleeding)^		^Fluid discharge^		^- Leukorrhea^	^(i) If vaginal discharge is associated with foul smell, green code is automatically assigned^
				^- Mucus plug discharge (“show”)^	^(ii) If it is associated with fever, yellow code is automatically assigned^
^Itching during pregnancy^			^Generalized^	^Localized^	^(i) If itching during pregnancy is associated with mild headache, green code is automatically assigned^
					^(ii) If it is associated with the following, yellow code is automatically assigned:^
					^- Persistent headache^
					^- Nausea^
					^- Vomit^
					^- Persistent epigastric pain^
					^- Visual disturbances^
					^- Dyspnea^
					^- BP > 140/90 mmHg^
					^- Reduction in fetal movements >=23 weeks GA^
^Fetal growth restriction/arrest^			^Fetal growth restriction/arrest >= 23 weeks GA^	^Fetal growth restriction/arrest < 23 weeks GA^	
^Cutaneous rash^	^With glottic edema^	^Dyspnea^	^Itching^	^Cutaneous rash^	
^Shock^	^State of shock^				
^Trauma^	^- Unintentional abdominal trauma^	^- Acute pain^			
	^- Unintentional trauma (not abdominal)^	^- Bleeding^			
	^- Beatings^	^- Abdominal pain^			
	^- Fall^	^- Amniotic fluid leakage^			
		^- Uterine contractions^			

**Table 2:** General population characteristics.

	2019	2020	*p*	Δ
General population	*n* = 1246	*n* = 611	<0.0001	−50.96%
Gynecological patients	336 (27.1%)	69 (11.3%)	<0.0001	−79.46%
Obstetrical patients	906 (72.9%)	541 (88.7%)		−40.28%
Multiparous women	625 (50.4%)	308 (52.4%)	0.951	−50.72%
Nationality				
Italian	851 (68.3%)	381 (62.4%)	0.006	−55.22%
Other nationalities	395 (31.7%)	230 (37.6%)		−41.77%
Self-administered therapy				
	137 (11%)	44 (7.2%)	0.005	−67.88%
Time in emergency room (min)				
Less than 30	106 (8.5%)	88 (14.4%)	0.004	−16.98%
From 30 to 60	318 (25.5%)	132 (21.6%)		−58.49%
From 60 to 90	332 (26.6%)	184 (30.1%)		−44.57%
From 90 to 120	188 (15.1%)	89 (14.6%)		−47.34%
More than 120	302 (24.2%)	118 (19.3%)		−60.92%
Discharge code				
Red	0 (0.0)	2 (0.3)	<0.0001	—
Yellow	37 (3.0)	48 (7.9)		+29.72%
Green	1032 (82.8)	493 (80.7)		−52.22%
White	177 (14.2)	68 (11.1)		−61.58%
LWBS	102 (8.2%)	2 (0.3%)	<0.0001	−98.30%
Hospitalization	214 (17.2%)	192 (31.6%)	<0.0001	−10.28%

**Table 3:** General population admission and discharge.

	Admission symptoms				Diagnosis at discharge			
	2019	2020	*p*	Δ	2019	2020	*p*	Δ
Bleeding	334 (26.9%)	180 (29.5%)	0.254	−46.10%	257 (20.6%)	76 (12.4%)	<0.0001	−70.42%
Pain	496 (39.9%)	131 (21.4%)	<0.0001	−73.58%	442 (35.5%)	83 (13.6%)	<0.0001	−81.22%
Distal genital tract symptoms	64 (5.1%)	22 (3.6%)	0.139	−65.62%	56 (4.5%)	18 (2.9%)	0.109	−67.85%
Urinary symptoms	63 (5.1%)	24 (3.9%)	0.278	−61.90%	78 (6.3%)	19 (3.1%)	0.004	−75.64%
Mammary symptoms	22 (1.8%)	11 (1.8%)	0.958	−50.00%	24 (1.9%)	10 (1.6%)	0.662	−58.33%
Fever	13 (1%)	5 (0.8%)	0.640	−54.54%	12 (1%)	2 (0.3%)	0.137	−83.33%
Gastrointestinal symptoms	30 (2.4%)	6 (1%)	0.036	−80.00%	64 (5.1%)	8 (1.3%)	<0.0001	−87.50%

**Table 4:** Obstetrical patients' characteristics.

Characteristics	2019 (*n* = 906)	2020 (*n* = 541)	*p*	Δ	
*Discharge code*					
Red	0 (0%)	2 (0.4%)	0.005	—	
Yellow	31 (3.4%)	47 (8.7%)		+51.61%	
Green	807 (89.1%)	449 (83%)		−44.36%	
White	68 (7.5%)	43 (7.9%)		−36.76%	
*Age*					
Less than 35 years	667 (73.7%)	393 (72.8%)	0.7	−41.07%	
From 35 to 50 years	238 (26.3%)	147 (27.2%)		−38.23%	
*Gestational age*					
From 0 to 12 weeks	219 (24.7%)	96 (18.9%)	<0.0001	−56.16%	
From 12 to 27 weeks	153 (17.3%)	52 (10.2%)		−66.01%	
From 27 to term	513 (58%)	361 (70.9%)		−29.62%	
Hospitalization	198 (21.9%)	188 (34.9%)	<0.0001	−05.05%	
LWBS	11 (1.2%)	1 (0.2%)	0.037	−90.90%	
*Minutes in emergency room and codes*					
<30	Red	0 (0%)	2 (2.4%)	<0.0001	—
	Yellow	3 (3.7%)	22 (26.8%)		+633.33%
	Green	70 (86.4%)	54 (65.9%)		−22.85%
	White	8 (9.9%)	4 (4.9%)		−0.5%
From 30 to 60	Yellow	10 (4.4%)	7 (6.0%)	0.039	−30.00%
	Green	201 (87.8%)	87 (75.0%)		−56.71%
	White	18 (7.9%)	22 (19.0%)		+22.22%
From 60 to 90	Yellow	10 (3.7%)	10 (5.7%)	0.454	00.00%
	Green	239 (89.5%)	154 (88.0%)		−35.56%
	White	18 (6.7%)	11 (6.3%)		−38.88%
From 90 to 120	Yellow	4 (3.0%)	3 (4.1%)	0.597	−25.00%
	Green	121 (90.3%)	67 (90.5%)		−44.62%
	White	9 (6.7%)	4 (5.4%)		−55.55%
More than 120	Yellow	4 (2.1%)	5 (5.3%)	0.018	−25.00%
	Green	176 (90.3%)	87 (92.6%)		−50.56%
	White	15 (7.7%)	2 (2.1%)		−86.66%

**Table 5:** Obstetrical patients' admission and discharge.

	Admission symptoms and signs				Diagnosis at discharge			
	2019	2020	*p*	Δ	2019	2020	*p*	Δ
Bleeding	230 (25.5%)	159 (29.4%)	0.1	−30.86%	163 (18%)	61 (11.3%)	0.001	−62.75%
Pain	313 (34.6%)	105 (19.4%)	<0.001	−66.45%	266 (29.4%)	71 (13.1%)	<0.0001	−73.30%
Distal genital tract issues	28 (3.1%)	9 (1.7%)	0.096	−67.85%	18 (2%)	4 (0.7%)	0.061	−77.77%
Urinary affections	26 (2.9%)	17 (3.1%)	0.768	−36.41%	34 (3.8%)	13 (2.4%)	0.161	−61.76%
Mammary symptoms	3 (0.3%)	4 (0.7%)	0.279	−25.00%	3 (0.3%)	4 (0.7%)	0.279	−25.00%
Fever	8 (0.9%)	4 (0.7%)	0.771	−50.00%	8 (0.9%)	2 (0.4%)	0.254	−87.27%
Gastrointestinal issues	25 (2.8%)	5 (0.9%)	0.18	−80.00%	55 (6.1%)	7 (1.3%)	<0.0001	−87.27%
Respiratory issues	14 (1.5%)	3 (0.6%)	0.091	−78.57%	14 (1.5%)	1 (0.2%)	0.013	−92.85%
Cardiac issues	5 (0.6%)	0 (0%)	0.083	−100.00%	4 (0.3%)	0 (0.0%)	0.202	−100.00%
Hypertensive disorders	32 (3.5%)	18 (3.3%)	0.837	−43.75%	47 (3.8%)	18 (2.9%)	0.221	−61.70%
Decreased fetal movements	50 (4.0%)	24 (3.9%)	0.517	−52.00%	45 (3.6%)	14 (2.3%)	0.08	−68.88%

**Table 6:** Obstetrical patients' specific admission and discharge.

	2019	2020	*p*	Δ
Uterine contractions	310 (24.9%)	219 (35.8%)	<0.0001	−29.35%
Labor/delivery	28 (2.3%)	129 (21.1%)	<0.0001	+360.71%
Vaginal discharge	102 (8.2%)	112 (18.3%)	<0.0001	+09.80%
PROM	99 (7.9%)	60 (9.8%)	0.175	−65.00%
Miscarriage	49 (3.9%)	46 (7.5%)	0.001	−06.12%
Threatened abortion	13 (76.5%)	0 (0.0%)	<0.0001	−100.00%
Threatened preterm labor	4 (23.5)	1 (0.1%)		−75.00%
Vomit-nausea	73 (8.1%)	24 (4.4%)	0.008	−68.49%
Blood tests and scheduled therapies	16 (1.8%)	1 (0.2%)	0.007	−93.75%
Regular pregnancy	242 (19.4%)	85 (13.9%)	0.002	−64.87%

**Table 7:** Gynecological patients' characteristics.

		2019	2020	*p*			Δ	
		*n* = 336	*n* = 69				−79.46%	
Minutes in emergency room and codes								
<30	Yellow	3 (12.0%)	0 (0.0%)	0.430			−100%	
	Green	16 (64.0%)	4 (66.7%)				−75.00%	
	White	6 (24.0%)	2 (33.3%)				−66.66%	
From 30 to 60	Yellow	1 (1.1%)	0 (0.0%)	0.246			−100%	
	Green	61 (69.3%)	9 (56.3%)				−85.24%	
	White	26 (29.5%)	7 (43.8%)				−73.07%	
From 60 to 90	Green	44 (68.8%)	4 (44.4%)	0.154			−90.90%	
	White	20 (31.3%)	5 (55.6%)				−75.00%	
From 90 to 120	Green	32 (59.3%)	8 (57.1%)	0.888			−75.00%	
	White	22 (40.7%)	6 (42.9%)				−72.72%	
More than 120	Yellow	1 (1.0%)	1 (4.2%)	0.158			—	
	Green	69 (65.7%)	18 (75.0%)				−73.91%	
	White	35 (33.3%)	5 (20.8%)				−85.71%	
Hospitalization		15 (4.5%)	4 (5.9%)	0.619			−73.33%	
LWBS		91 (27.1%)	1 (1.4%)	<0.0001			−98.90%	

	Admission symptoms and signs				Diagnosis at discharge			
	2019	2020	*p*	Δ	2019	2020	*p*	Δ

Bleeding	104 (31.1%)	21 (30.4%)	0.90	−79.80%	94 (28%)	14 (20.3%)	0.189	−85.10%
Pain	179 (53.6%)	26 (37.7%)	0.016	−85.47%	173 (51.5%)	12 (17.4%)	<0.0001	−93.06%
Distal genital tract issues	36 (10.7%)	13 (18.8%)	0.60	−63.88%	38 (11.3%)	14 (20.3%)	0.042	−63.15%
Urinary issues	36 (10.7%)	7 (10.1%)	0.883	−80.55%	43 (12.8%)	6 (8.7%)	0.342	−86.04%
Mammary issues	19 (5.7%)	7 (10.1%)	0.167	−63.15%	21 (6.3%)	6 (8.7%)	0.459	−71.42%
Fever	5 (1.5%)	1 (1.4%)	0.976	−80.00%	4 (1.2%)	0 (0%)	0.364	−100%
Menstrual disorders	20 (6%)	6 (8.7%)	0.398	−70.00%	47 (14%)	4 (5.8%)	0.062	−91.48%
Pelvic organ prolapse	4 (1.2%)	0 (0%)	0.362	−100%	4 (1.2%)	0 (0%)	0.364	−100%
Gastrointestinal issues	5 (1−5%)	1 (1.4%)	0.976	−80.00%	9 (2.7%)	1 (1.4%)	0.55	−88.8
Postsurgical complications	—				7 (2.1%)	0 (0%)	0.228	−100%
Fibromatous uterus	—				3 (0.9%)	1 (1.4%)	0.671	−66.66%
Malignant neoplasm	—				0 (0%)	1 (1.4%)	0.027	—
Ovarian cysts	—				1 (0.3%)	3 (4.3%)	0.02	+200%
Sexual abuse	—				2 (0.6%)	1 (1.4%)	0.452	−50.00%

**Table 8:** Time-related obstetric and gynecological complications.

Time-related complications	2019	2020
Small for gestational age childbirths	26	22
Intrauterine growth restriction	1	0
Stillbirth	1	0
Moderate preeclampsia	10	7
Severe preeclampsia/eclampsia/HELLP syndrome	3	2
Placental abruption	2	5
Preterm birth	43	45
Neonatal asphyxia	14	10
Miscarriage	8	5
Hemoperitoneum	4	1

## Data Availability

The data can be requested by contacting Dr. Roberta Amadori (roberta.amadori@uniupo.it).
